# A giant arachnoid cyst: Is it an innocent bystander?

**DOI:** 10.1002/ccr3.3004

**Published:** 2020-06-02

**Authors:** Soonwoong Hong, John‐Ross D. Clarke, Lawrence Beck

**Affiliations:** ^1^ Department of Internal Medicine Yale‐New Haven Health/Bridgeport Hospital Bridgeport CT USA

**Keywords:** arachnoid cyst, computed tomography, Galassi classification, magnetic resonance imaging

## Abstract

Arachnoid cysts vary in their size and location. Large cysts may cause symptoms requiring surgery. It is important to assess whether patients with arachnoid cysts and neurologic symptoms can benefit from such surgical interventions.

## Graphical abstract

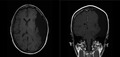

## 1 CASE DESCRIPTION

A 47‐year‐old woman presented with syncope. Magnetic resonance imaging of the brain and computer tomography of the head revealed an exceptionally large arachnoid cyst with mass effect. While only approximately 5% of arachnoid cysts cause symptoms, it remains important to assess whether an arachnoid cyst can cause a patient's symptomatology.

**FIGURE 1:**
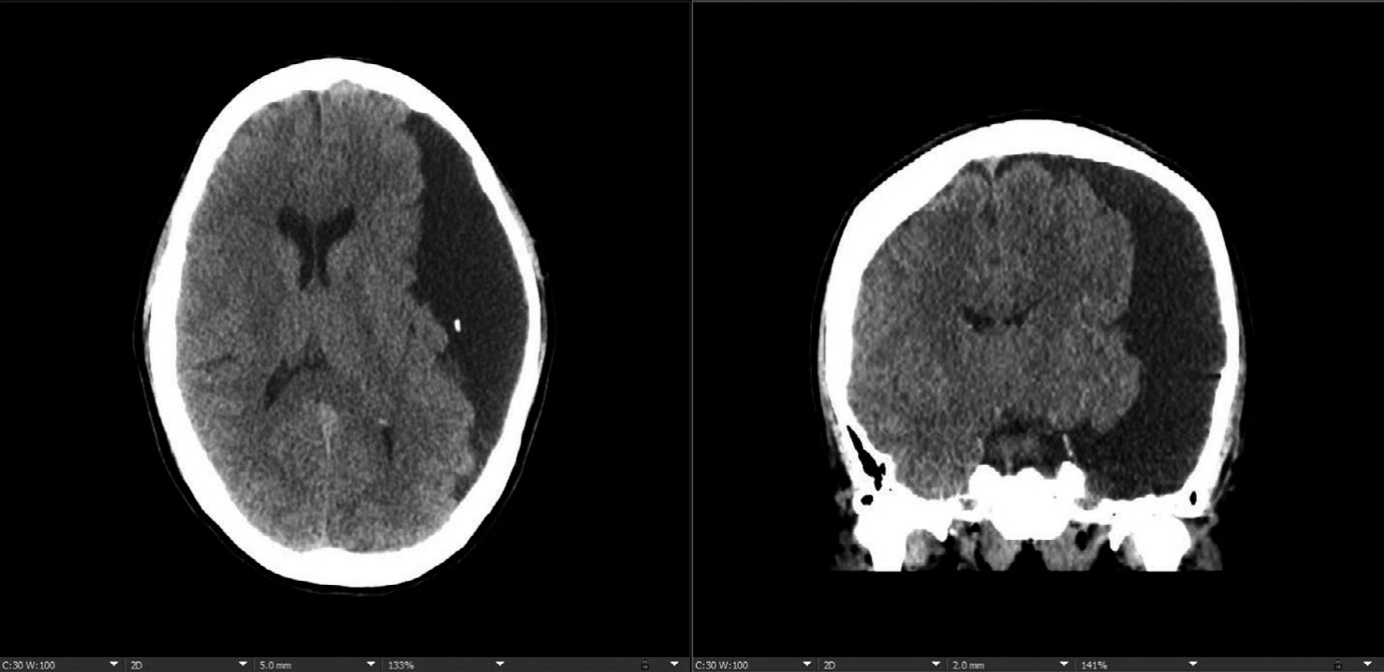
Computed tomography images of the head in axial (left) and coronal views (right)—showing large CSF density extra‐axial fluid collection in the left hemisphere measuring 12 cm × 11 cm × 4.5 cm in size along the left cerebral convexity. There is no communication with the ventricular system with smooth scalloping of the calvarium on the left side

**FIGURE 2:**
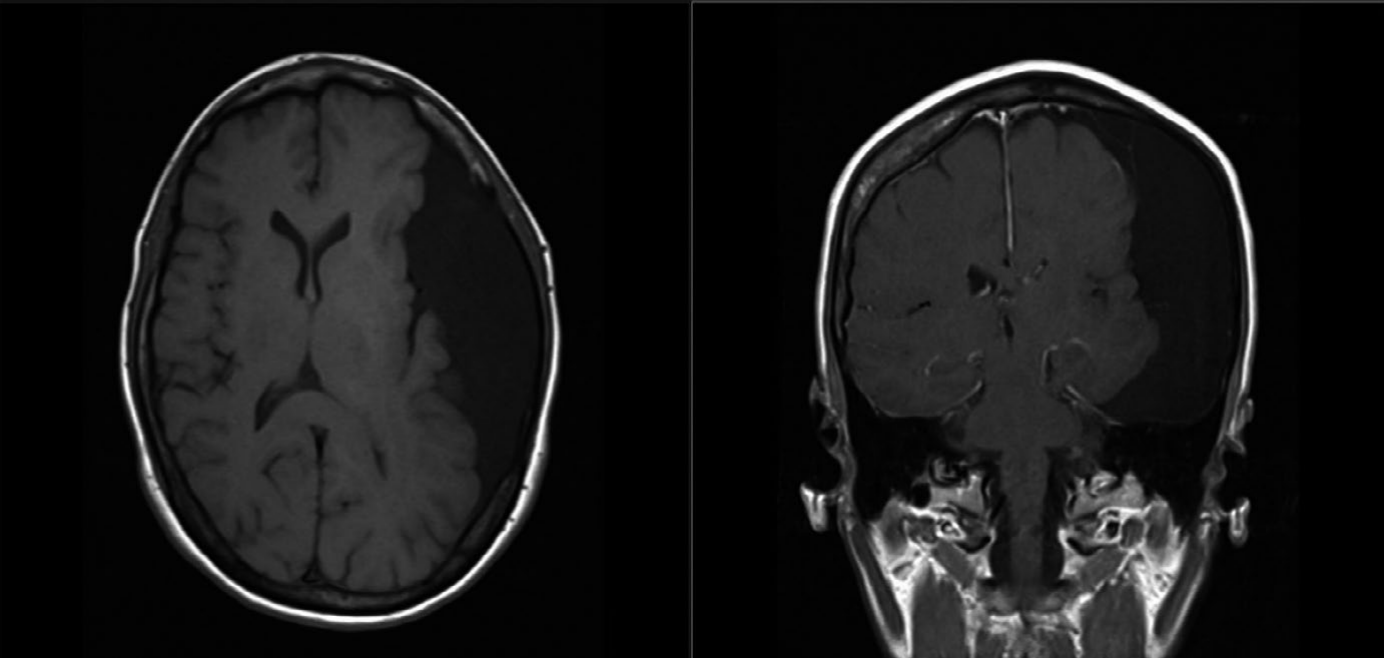
Magnetic resonance images of the head in axial (left) and coronal views (right)—showing large left‐sided extra‐axial collection with some internal septations (without postcontrast enhancement), that exerts mass effect on the adjacent left cerebral hemisphere and a rightward midline shift of about 3 mm

A 47‐year‐old woman presented with syncope. Neurological examination was unremarkable except for midline‐splitting left‐sided sensory loss to all modalities, suggestive of functional sensory loss. Computed tomography of the head (Figure 1) and magnetic resonance imaging (MRI) of the brain (Figure 2) revealed a 12 × 11 × 4.5‐cm left‐sided extra‐axial collection with mass effect. Electroencephalography findings were normal. She was evaluated by neurosurgery, who suggested the cyst to be an incidental finding unrelated to her symptomatology; thus, nonsurgical management was recommended.

Arachnoid cysts are intracranial cerebrospinal fluid‐filled sacs covered by the arachnoid membrane and are incidentally found in up to 1.4% of persons who undergo brain MRI. Only approximately 5% of patients with arachnoid cysts have cyst‐related neurological symptoms.^1^ The Galassi classification is used to classify arachnoid cysts in the middle cranial fossa:^2^ Type I cysts are typically small and asymptomatic, located in the anterior middle cranial fossa. Type II cysts stretch superiorly along the Sylvian fissure and can displace the temporal lobe. Type III cysts are exceptionally large, taking up the entire middle cranial fossa, displacing the temporal, parietal, and frontal lobes.^2^ The cyst in this case can be classified as type III. Treatment depends on whether patients are symptomatic. Conservative treatment involves serial imaging, while surgical options include craniotomy, cystoperitoneal shunt placement, endoscopic fenestration, or stereotactic aspiration.

## CONFLICT OF INTEREST

There are no conflicts of interest to disclose.

## AUTHOR CONTRIBUTION

SH: experienced current case, acquisition of data, participated in drafting, and revising the article. JDC: made substantial contribution to conception, acquisition of data, and participated in drafting the article. LB: made substantial contribution to conception and gave final approval of the version to be submitted and any revised version.
